# Acceptability of online exercise-based interventions after breast cancer surgery: systematic review and narrative synthesis

**DOI:** 10.1007/s11764-020-00931-6

**Published:** 2020-09-15

**Authors:** Mariya B. Sotirova, Eilís M. McCaughan, Lucia Ramsey, Carrie Flannagan, Daniel P. Kerr, Sean R. O’Connor, Nicole E. Blackburn, Iseult M. Wilson

**Affiliations:** 1 Institute of Nursing and Health Research, Ulster University, Jordanstown, Northern Ireland UK; 2 Institute of Nursing and Health Research, Ulster University, Coleraine, Northern Ireland UK; 3 Centre for Public Health, Queen’s University Belfast, Belfast, Northern Ireland UK; 4 School of Nursing and Midwifery, Queen’s University Belfast, Belfast, Northern Ireland UK

**Keywords:** Internet, Adherence, Cancer, Exercise, Rehabilitation, Surgery

## Abstract

**Purpose:**

eHealth and mHealth approaches are increasingly used to support cancer survivors. This review aimed to examine adherence, acceptability and satisfaction with Internet-based self-management programmes for post-surgical cancer rehabilitation and to identify common components of such interventions.

**Methods:**

Nine electronic databases were searched from inception up to February 15, 2020, for relevant quantitative and qualitative studies evaluating Internet-based cancer rehabilitation interventions. Studies were required to include an exercise or physical activity–based self-management intervention and a measure of adherence, acceptability or user satisfaction with the programme. Two independent reviewers performed all data extraction and quality assessment procedures. Data were synthesized using a narrative approach.

**Results:**

Six hundred ninety-six potential papers were identified and screened. Eleven met the inclusion criteria. Interventions had wide variations in levels of adherence, but the majority were reported as being acceptable to the users. Increased acceptability and user satisfaction were associated with interventions which were seen as time and cost-efficient, requiring acquisition of minimal or no new skills, which used coherent language, or which provided tailored information. The majority contained behaviour change components such as goal setting.

**Conclusions:**

Despite high levels of heterogeneity between studies, Internet-based approaches may be an acceptable method for the delivery of self-management interventions in post-surgical cancer rehabilitation.

**Implications for Cancer Survivors:**

There is a need for further studies exploring factors associated with increased user engagement and usage of digital interventions in cancer rehabilitation settings. These findings should be used to help develop interventions prior to testing their effectiveness in adequately powered randomized controlled trials.

## Introduction

Despite increasing incidence, cancer survival rates have doubled in the past 40 years [1]. More than 40% of people undergo surgical interventions as part of their primary cancer treatment [2] and while many people are able to return to pre-diagnosis occupations and lifestyle [3–5], treatment-associated side effects are a common occurrence [6]. This includes functional and musculoskeletal issues such as loss of muscular and cardiac fitness, fatigue, impaired motor sensory function and lymphoedema [6]. Cancer multidisciplinary rehabilitation is found by the literature [7–9] to minimize these effects in its capacity as a key element of the care that cancer patients receive, aiming to minimize long-term complications, reducing hospital re-admissions and improving quality of life [7–9]. Cancer rehabilitation assists individuals to achieve the best possible physical, psychological, social and vocational outcomes [10]. A multidisciplinary team approach which anticipates the needs of cancer survivors in a timely, coordinated and continuous manner from the time of diagnosis is recommended [10]. Worldwide policy drivers for patient empowerment during cancer treatments emphasize the need for self-management and person-centred interventions to address unmet care needs [11]. Studies suggest that approximately 40% of patients report at least one unmet need for rehabilitation services in the immediate recovery period and in the longer term [12–14]. A large-scale cross-sectional survey also showed that 63% of cancer survivors had a need for at least one type of rehabilitative service, with physiotherapy and physical training being the most often required (43% and 34%, respectively) [15].The Internet is a powerful medium for providing accessible and low-cost resources to address unmet support needs in cancer survivorship. Although increasing, the number of these resources is relatively small and there is minimal evidence that describes users’ experience of accessing them [16, 17]. Engagement with interventions, facilitators and barriers to their use and users’ views on their acceptability, therefore, needs further examination [16–18]. Yardley and colleagues [18] suggest there is a clear distinction between effective engagement with an online intervention which leads to desired outcomes and behavioural change, and a minimal level of engagement, which might not necessarily effect change. Further evidence suggests a number of factors are associated with poor user engagement. This includes the provision of standard information instead of more specialist support and personalization of information [19, 20]. Engagement can, however, be limited by barriers such as lack of experience with using online resources and by usability issues [21, 22]. To inform future research in this area, the aim of this review was to comprehensively examine adherence, acceptability and satisfaction with exercise-based online self-management programmes for post-surgical cancer rehabilitation and to identify common components of such interventions.

## Methods

### Study design and search strategy

**Fig. 1:**
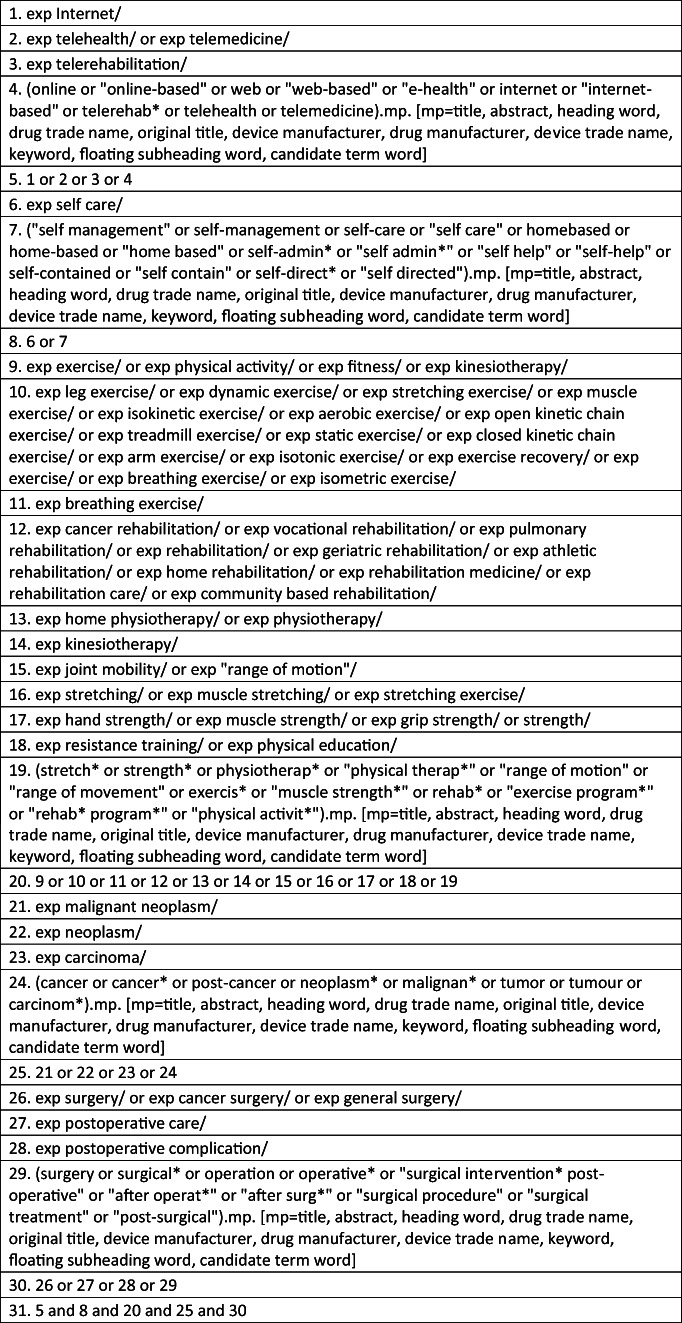
OVID-EMBASE search strategy

**Fig. 2:**
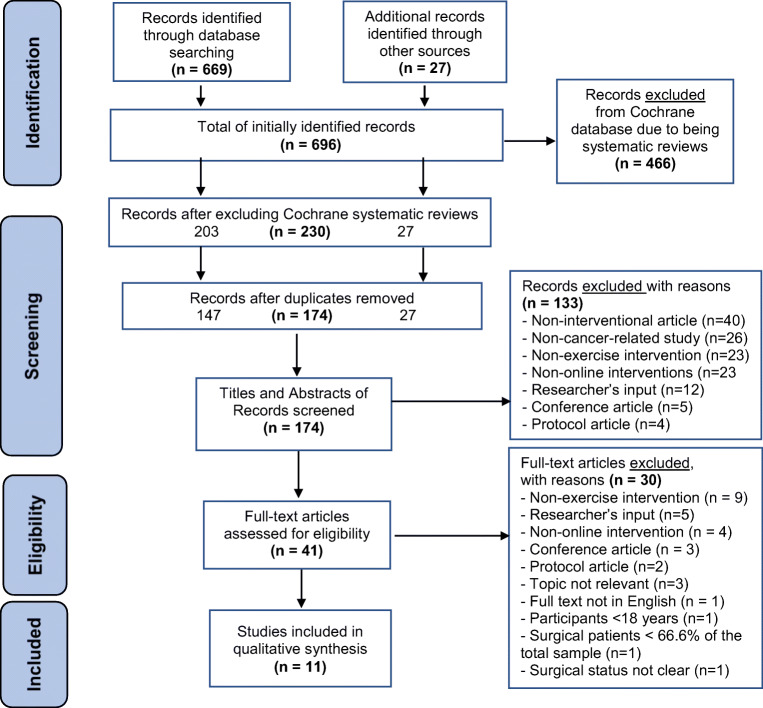
Study selection process with exclusion reasons

This systematic review (PROSPERO registration number: CRD42018107411) was conducted using a predefined protocol developed according to the recommendations of the Cochrane Collaboration Handbook [23] and the Preferred Reporting Items for Systematic Reviews and Meta-analysis (PRISMA) guidelines [24]. Nine electronic databases (The Allied and Complementary Medicine Database (AMED), The Cochrane Library, The Cumulative Index to Nursing and Allied Health Literature (CINAHL), Excerpta Medica Database (EMBASE), Medical Literature Analysis and Retrieval System Online (MEDLINE), Physiotherapy Evidence Database (PEDro), ProQuest Medical Library, Pubmed and Scopus) were searched from inception to February 15, 2020. Figure 1 presents a copy of the search syntax for the Ovid-EMBASE database to facilitate replication of the search. In order to identify unindexed articles in the searched databases, grey literature was searched using Google scholar, as well as manual searches of the reference lists of relevant articles in the field. The search strategy included a list of concepts (Internet, self-management, exercise, cancer, surgery, response to intervention), with an extensive list of associated keywords and MeSH terms (Table 1). The “explode” command, the truncation symbol (*) and Boolean terms (AND, OR) were applied in order to combine the different search concepts. Two independent reviewers (MS and IW) screened identified titles and abstracts before screening full-text copies of potentially relevant articles based on inclusion and exclusion criteria outlined below. Final agreement on study inclusion was agreed by both reviewers with a third reviewer (LR) consulted to resolve any disagreements (see Fig. 2 for PRISMA flow diagram).

**Table 1:** Search terms, concepts and medical headings

Search concepts	Keywords	MeSH
1) Internet	Internet or “Internet-based” or online or “online-based” or web or “web-based” or “e-health” or comput* or PC or website or mobile or ehealth or mhealth or “m-health” or telemedicine or telehealth or telerehab* or teletherap*	Internet E-health Telerehabilitation Telehealth Telemedicine
2) Self-management	“self management” or self-management or self-care or “self care” or homebased or home-based or “home based” or self-admin* or “self admin*” or “self help” or “self-help” or self-contained or “self contain” or self-direct* or “self directed”	Self-care Self-management “Self-directed learning as topic”
3) exercise	stretch* or strength* or physiotherap* or “physical therap*” or “range of motion” or “range of movement” or exercis* or “muscle strength*” or rehab* or “exercise program*” or “rehab program*” or exercise or “physical activit*”	Exercise therapy Physical therapy Rehabilitation Physical activity/exercise Muscle stretching exercises Resistance training Range of motion, articular Activities of daily living Early ambulation
4) Cancer	cancer or cancer* or post-cancer or neoplasm* or malignan* or tumour or carcinom* or oncolog*	Neoplasms Carcinoma
5) Surgery	surgery OR surgical* OR operation OR operative* OR “surgical intervention” OR “postoperative” OR “after operat*” OR “after surg*” OR “surgical procedure” OR “surgical treatment” OR “post-surgical”	General surgery Postoperative complications/surgical procedures, operative/surgery, operative and postoperative care
6) Response to intervention	accept* or adher* or barrier* or facilitat* or preference* or reaction or satisfact* or uptake or usab*	Treatment adherence and compliance, personal satisfaction, patient acceptance of healthcare, health knowledge, attitudes, practice, patient preference, patient attitude

### Inclusion and exclusion criteria

To be included, studies were required to meet the following criteria:

1. Quantitative or qualitative study design

- Included adult participants (aged 18 or over) with at least 2/3 of the study sample having received surgical intervention for any type of cancer
- Included an Internet-based, self-management intervention which included any form of exercise or physical activity, e.g., walking cycling, etc.
- Included at least one measure related to adherence, acceptability and/or satisfaction with the intervention

As the majority of the authors were not multilingual and it was beyond resources of the team to involve a translator, it was decided to restrict the studies examined to those published in English.

### Definitions

For the purposes of this article, the following definitions were used in order to avoid ambiguity in terms of defining Internet-based interventions, adherence, acceptability and satisfaction.

#### Internet interventions

Bennett and Glasgow [25] define these as “Systematic treatment/prevention programs, usually addressing one or more determinants of health (frequent health behaviours), delivered largely via the Internet (although not necessarily exclusively Web-based), and interfacing with an end user. These interventions are typically highly structured, mostly self-guided, interactive, and visually rich, and they may provide tailored messaging based on end-user data”.

#### Adherence to a treatment modality

Adherence has been defined by Kelders and colleagues [26] as “The extent to which the patient’s behaviour matches the recommendations that have been agreed upon with the prescriber”. According to the definition we used and as per the context of this definition provided by its authors, “the patient’s behaviour” is considered the usage or the absence of usage of the intervention and whether or not it matches the intended intervention usage that is recommended by the intervention creators [26].

#### Treatment acceptability

Sekhon and colleagues [27] define acceptability as “A multi-faceted construct that reflects the extent to which people delivering or receiving a healthcare intervention consider it to be appropriate, based on anticipated or experienced cognitive and emotional responses to the intervention”. For the purposes of this article, this definition describes emotional or cognitive responses to an intervention that may or may not involve usage of the intervention. As per the given context of this definition by its authors, it includes the users’ perceptions of treatment acceptability for both: before and as a result of participating or using a treatment intervention [27].

#### User satisfaction with web-based health interventions

Bob and colleagues [28] define user satisfaction with web-based health interventions as “Satisfaction is a user’s evaluation of the received Web-based intervention”. For the purposes of this article, the definition for *satisfaction* does not describe, and therefore distinguishes itself, from the emotional or cognitive reaction to an intervention. This definition only describes the evaluation processes that the intervention users might undergo during or after intervention usage in order to approve or disapprove a given intervention [28].

#### Self-management interventions

“Interventions that aim to equip patients with skills to actively participate and take responsibility in the management of their chronic condition. This includes knowledge acquisition, and a combination of at least two of the following: (1) stimulation of independent sign and/or symptom monitoring; (2) medication management; (3) enhancing problem-solving and decision-making skills for treatment or disease management; (4) or changing physical activity, dietary and/or smoking behaviour” [29].

### Data extraction and methodological quality assessment

Predefined data extraction tables were used to summarize study designs and main characteristics (Table 2), participant characteristics (Table 3), types and features of interventions (Table 4) and the main study findings (Table 5). Methodological quality was assessed using the **s**tandard **q**uality **a**ssessment **c**riteria for **e**valuating **p**rimary **r**esearch **p**apers from a **v**ariety of **f**ields by Kmet and colleagues [30] which consists of two separate quality assessment scales for qualitative and quantitative studies (Tables 6 and 7). The quality of the studies was rated according to the scoring that Lee et al. [31] and Maharaj and Harding [32] used in their similarly designed reviews. Study quality was rated according to accepted scoring methods and cut-offs with summary scores > 80% defined as “strong”, 71–79% as good, 50–70% as adequate, and scores of < 50% indicating **“**poor**”** or limited quality. Data extraction and quality assessment w**ere** conducted by at least two independent reviewers (MS and IW or LR) and inter-rater level of agreement between the reviewers was evaluated using Cohen’s **k**appa [33] and Cohen’s **w**eighted **k**appa values [34]. Studies were not excluded from the synthesis based on quality scores, which were used to interpret **the** findings of the review.

**Table 2:** General characteristics of the included studies

Study/author/year/country	Study aims	Study design	Data collection tools
Cnossen et al. (2016) [43] The Netherlands	Aim: To explore the feasibility of a self-care education programme, by measuring intervention usage, uptake and the end-user satisfaction, in addition to secondary care	Quantitative study A single-group cross-sectional feasibility and satisfaction study design	Study-specific survey 10-point Likert scale A study-specific questionnaire
Foster et al. (2016) [38] United Kingdom	Aim: To assess the proof of concept of the RESTORE web-based intervention	Mixed-methods study A multicentre parallel-group two-armed exploratory randomized controlled trial with qualitative process evaluation	Semi-structured telephone interviews Data usage Questionnaires
Harder et al. (2017) [44] United Kingdom	Aim: To present the development process of the bWell app and the preliminary results of early user testing	Qualitative focus group study with preliminary user testing	Focus group discussions
Kanera et al. (2016) [39] The Netherlands	Aim: To assess the effects of the web-based life after cancer (KNW) intervention on the outcomes: physical activity, diet and smoking 6 months after using the KNW intervention	Quantitative study A randomized controlled trial	Online self-report questionnaires Data usage Login data
Kanera et al. (2017) [36] The Netherlands	Aim: To assess the long-term effects of the web-based life after cancer (KNW) intervention on the outcomes for moderate physical activity and vegetable consumption at 12 months after using the KNW intervention in order to track maintenance of these 2 outcomes between 6 months post baseline and 12 months	Quantitative study A randomized controlled trial	Online self-report questionnaires Data usage Login data
Lee et al. (2013) [46] Republic of Korea	Aim: To design and develop a web-based self-management diet and exercise intervention for cancer survivors which is based on the trans-theoretical model and to formally evaluate the intervention	A mixed-method qualitative and quantitative intervention development and formal evaluation study	Qualitative semi-structured interviews Questionnaires with 7-point scale Intervention usage
Lee et al. (2014) [40] South Korea	Aim: To investigate if the web-based self-management exercise and diet intervention (WSEDI) for breast cancer survivors based on the trans-theoretical model is feasible and having a primary effect on improving the quality of dietary behaviours and exercising.	Quantitative study A 12-week pilot randomized controlled trial with a control group	Self-reported online surveys 7-day exercise diary 3-day dietary recall Cancer-specific questionnaires Intervention usage
Melissant et al. (2018) [41] The Netherlands	Aim: To evaluate the feasibility of the Oncokompas intervention among breast cancer survivors, featuring the breast cancer module	Quantitative study A pre-test-post-test feasibility study	Semi-structured interviews Pre- and post-intervention surveys Consultation with oncology nurse Intervention usage
Myall et al. (2015) [45] United Kingdom	Aim: To explore the amount of work that the participants in the RCT related to this intervention were required to do	An in-depth qualitative process evaluation study	Semi-structured telephone interviews
Paxton et al. (2017) [42] United States	Aim(s): 1. To investigate if participants from the physical activity (PA) group would have greater improved moderate to physical activity level than those participants randomized in the Dietary group 2. To investigate if participants from the dietary group would have greater improved fruit and vegetable consumption than those participants randomized in the PA group	Quantitative study A randomized parallel-group feasibility study	Web-based survey consisting of 5-point Likert scaled questions Web-based survey: a yes/no question Web-based survey with open-ended questions Website usage tracking
Willems et al. (2017) [37] The Netherlands	Aim: To present the short-term effects of the KNW intervention on QoL, anxiety, depression and fatigue	Quantitative study A randomized controlled trial	Self-report questionnaires Modules usage

**Table 3:** Sample characteristics across the studies

Study/author/year	Sample size	*n* = intervention group/mean age (SD)/gender/% received surgery	*n* = control group/mean age (SD)/gender/% received surgery	Type of cancer/adjuvant treatment (yes/no/unknown)	Time since surgery/treatment (min/max)
Cnossen et al. (2016) [43]	*n* = 38	*n* = 38 Mean age: 65 Male *n* = 29 (76%) Female *n* = 9 (24%) 100% Total laryngectomy	None	Head and neck cancer Radio-/chemotherapy: unknown	Between 3 months to 2 years prior to the study
Foster et al. (2016) [38]	*n* = 159	*n* = 83 Mean age: 58.1 (10.7) Male *n* = 22 (26.5%) Female *n* = 61 (73.5%) Surgery: 85.5% (*n* = 71)	*n* = 76 Mean age: 57.5 (9.1) Male *n* = 15 (19.7%) Female *n* = 61 (80.3%) Surgery: 84.2% (*n* = 64)	Various cancer types Radio-/chemotherapy: no	No minimal time threshold since surgery or treatment 5-year maximum period since diagnosis
Harder et al. (2017) [44]	*n* = 13	Phase 1 *n* = 9 Mean age: 52.3 Male *n* = 0 Female *n* = 9 (100%) Surgery: 100% (*n* = 9)	Phase 2 *n* = 4 Mean age: between 51 and 58 years Male *n* = 0 Female *n* = 4 (100%) Surgery: 100% (*n* = 4)	Breast cancer Radio-/chemotherapy: yes	Not stated
Kanera et al. (2016) [39]	*n* = 462	*n* = 231 ITT-analysed Mean age: 55.6 (11.5) Male *n* = 48 (20.8%) Female *n* = 183 (79.2%) Surgery: 83.5% (*n* = 193)	*n* = 231 ITT-analysed Mean age: 56.2 (11.3) Male *n* = 45 (19.5%) Female *n* = 186 (80.5%) Surgery: 80.6% (*n* = 186)	Various cancer types Radio-/chemotherapy: No	A minimum of 4 weeks since surgery
Kanera et al. (2017) [36]	*n* = 462	*n* = 231 ITT-analysed Mean age: 55.6 (11.5) Male *n* = 48 (20.8%) Female *n* = 183 (79.2%) Surgery: 83.5% (*n* = 193)	*n* = 231 ITT-analysed Mean age: 56.2 (11.3) Male *n* = 45 (19.5%) Female *n* = 186 (80.5%) Surgery: 80.6% (*n* = 186)	Various cancer types Radio-/chemotherapy: no	A minimum of 4 weeks since surgery
Lee et al. (2013) [46]	*n* = 76	Development phase *n* = 46 No other information is provided by the authors	Evaluation phase *n* = 30 Mean age: 41.5 (6.3) Male: not stated Female: not stated Surgery: 100% (*n* = 30)	Breast cancer Radio-/chemotherapy: yes	No upper time limit since initial cancer diagnosis or treatment
Lee et al. (2014) [40]	*n* = 59	*n* = 30 (after randomization, before dropouts) Mean age: 41.5 (6.3) Female *n* = 30 (100%) Surgery: 100% (*n* = 30)	*n* = 29 (after randomization, before dropouts) Mean age: 43.2 (5.1) Female *n* = 29 (100%) Surgery: 100% (*n* = 29)	Breast cancer Radio-/chemotherapy: no	No minimal time threshold since surgery or treatment
Melissant et al. (2018) [41]	*n* = 68	*n* = 68 (participants left after dropouts. Before dropouts: *n* = 76 post-baseline) Mean age: 56 (12) Male: not stated Female: not stated Surgery: 100% (*n* = 68)	None	Breast cancer Radio-/chemotherapy: no	A minimum of 4 weeks since surgery
Myall et al. (2015) [45]	*n* = 19	*n* = 8 (after consent) Mean age of total sample: *n* = 14 < 60 years *n* = 5 > 60 years Gender of total sample: Male: 4 Female: 15 Surgery for the total Received by: *n* = 16 No surgery: n = 1 Missing: n = 2	*n* = 11 (after consent) Mean age of total sample: *n* = 14 < 60 years *n* = 5 > 60 years Gender of total sample: Male: 4 Female: 15 Surgery for the total Received by: n = 16 No surgery: n = 1 Missing: n = 2	Various cancer types Radio-/chemotherapy: no	No minimal time threshold since surgery or treatment 5-year maximum period since diagnosis
Paxton et al. (2017) [42]	*n* = 71	*n* = 34 Randomized after consent, ITT, before dropouts Mean age: 52.7 (8.4) Male: not stated Female: not stated Surgery: *n* = 31 (91%)	*n* = 37 Randomized after consent, ITT, before dropouts Mean age: 51.8 (8.9) Male: not stated Female: not stated Surgery: *n* = 36 (97%)	Breast cancer Radio-/chemotherapy: no	No upper time limit since initial cancer diagnosis or treatment
Willems et al. (2017) [37]	*n* = 462	*n* = 231 ITT-analysed Mean age: 55.6 (11.5) Male: *n* = 48 (20.8%) Female: *n* = 183 (79.2%) Surgery: 83.5% (*n* = 193)	*n* = 231 ITT-analysed Mean age: 56.2 (11.3) Male: *n* = 45 (19.5%) Female: *n* = 186 (80.5%) Surgery: 80.6% (*n* = 186)	Various cancer types Radio-/chemotherapy: no	A minimum of 4 weeks since surgery

**Table 4:** Breakdown of the features of the included interventions

Feature	Study (author/year/name of intervention/duration/web/app based)										
	Cnossen et al. (2016) [43] ITwC 2 weeks Web	Myall et al. (2015) [45] RESTORE 6 weeks Web	Foster et al. (2016) [38] RESTORE 6 weeks Web	Kanera et al. (2016) [39] KNW 6 months (26 weeks) Web	Kanera et al. (2017) [36] KNW 6 months (26 weeks) Web	Willems et al. (2017) [37] KNW 6 months (26 weeks) Web	Lee et al. (2013) [46] WSEDI 12 weeks Web	Lee et al. (2014) [40] WSEDI 12 weeks Web	Melissant et al. (2018) [41] Oncokompas 1 week Web	Paxton et al. (2017) [42] ALIVE 3 months 12 weeks Web + Email	Harder et al. (2017) [44] 8 weeks App
Action/coping plan				✓			✓			✓	✓
Assistance from researchers during the interventional period	✓			✓							
Automated emails		✓		✓						✓	
Automated phone calls										✓	
Automated SMS text messages							✓				
Automated tailored (specific) advice on which intervention parts to undertake				✓					✓		
Automated tailored (specific) progress feedback		✓		✓			✓		✓	✓	✓
Diary		✓					✓				✓
Exercise activity calendar	✓										✓
Exercise programme/information	✓	✓		✓			✓		✓	✓	✓
Exercise workbook	✓										
FAQs											✓
Forum for peer support				✓						✓	
Goal setting feature(s)		✓		✓			✓			✓	
Images/visual graphics	✓	✓		✓			✓		✓	✓	✓
Instructions on how to use the intervention				✓			✓		✓		✓
Online consent and/or baseline assessment		✓		✓					✓	✓	
Personal page				✓						✓	✓
Photos	✓										✓
Printables									✓		
Reminders				✓			✓			✓	✓
Restricted online login access with a passcode	✓	✓		✓			✓		✓	✓	✓
Rewards										✓	
Self-care information	✓			✓					✓		
Self-care skills education	✓										
Self-evaluation (of progress)				✓			✓		✓	✓	✓
Separate modules available at all times	✓			✓			✓		✓		✓
Separate modules released consecutively		✓								✓	
Tailored educational information				✓			✓		✓	✓	✓
Theoretical framework-based		✓	✓	✓			✓		✓	✓	
“Traffic light” notification/advice system				✓					✓		
Troubleshooting guides for symptom management	✓			✓					✓		
Video animations	✓										
Video demonstrations	✓										✓
Videos with other patients’ stories		✓		✓							
Videos with HCPs and/or educational info and advice				✓							
Web links for further information		✓		✓					✓		✓

**Table 5:** Main findings related to primary, secondary and effectiveness outcomes across the studies

Study/authors/year/design/follow-up	Main findings for primary outcomes of interest: adherence, acceptability and satisfaction	Main findings for secondary outcomes of interest: barriers and facilitators, moderating factors, suggestions for improvement of interventions, self-efficacy for fatigue self-management, patient activation measure, perceived ease of use, perceived effectiveness of the intervention	Main findings for effectiveness outcomes related to physical activity and dieting behaviours, self-efficacy, smoking cessation behaviours
Cnossen et al. (2016) [43] A single-group cross-sectional feasibility and satisfaction study design Follow-up: at 2 weeks	Satisfaction: - Satisfaction with the overall ITwC intervention: 84% -User-friendliness: 74% -Overall satisfaction with the self-care programme: 66% - Net promoter score: 25% would promote 20% would criticize = + 5% positive	1) Uptake rate: 73% 2) Measured by login: *n* = 38 (completers) out of *n* = 55 who consented and completed baseline measurements (69%) < 60 mins: 55% 60–90 min: 29% > 90 mins: 16% 3) Statistical association between satisfaction with the intervention programme and: - Level of education *p* = 0.004 - Health literacy skills *p* = 0.038 No significant association between satisfaction and: - Gender (*p* = 0.46) - Age (*p* = 0.50) - Marital status (*p* = 1.00) - Employment status (*p* = 1.00) - Internet literacy (*p* = 0.10) - Internet usage (*p* = 0.06) - Treatment modality (*p* = 0.46) - Time since TL (*p* = 1.00) - QoL (*p* = 0.75)	
Foster et al. (2016) [38] A multicentre parallel-group two-armed exploratory randomized controlled trial with qualitative process evaluation Follow-ups: at 6 and 12 weeks	Acceptability: 36%—total attrition rate; half of the participants having access to both: RESTORE and leaflet preferred to access the RESTORE programme; the other half preferred the leaflet Adherence to the intervention: 71% adherence to intervention (logged onto compulsory sessions 1 and 2 and at least one other optional; most visited optional session (51% of the participants): “Work and home life”; least visited optional session (27% of the participants): “Talking to others”	Feasibility: 41% of eligible participants consented to the study Randomized after consent and data collected at T0: *n* = 163	Change in perceived self-efficacy for fatigue self-management: Improved fatigue self-efficacy at T1 in RESTORE group compared with the control group (*p* = 0.09, group effect: 0.514)
Harder et al. (2017) [44] Phase 1: A qualitative focus group design study Phase 2: Preliminary user testing Follow-up: at 8 weeks	Findings from the developmental phase Five main themes identified within the first focus group discussion: 1) “Awareness of importance of exercises” 2) “Awareness that exercises are ongoing” 3) “Lacking or inconsistent advice” 4) “Gaps in care pathway and follow-up” 5) “Need for more directions or physiotherapy”	Second focus group discussion: - Very positive overall feedback - Suggestions for 1 × rating a day with colours - Suggestion for inclusion of a diary function - Suggestions for inclusion of Information section and FAQs section - Suggestions for models in demo videos to be of various ages and casually looking	Findings from the user testing phase User testing showed: - Ease of navigating - No technical issues - Coherent content, text and font of text Participants gave: Very positive feedback on: -The demo videos - The reminder feature of the app Somewhat beneficial feedback on: - Self-monitoring - The self-rating feature - Visual graphics of the app Beneficial feedback on: - Graded exercising tasks - Different exercising stages of the programme Overall feedback: -Intervention promoted motivation -Intervention was engaging −4.6/5 definite recommendation to other BC survivors recovering from a surgery for BC Intervention usage: all participants used it from almost daily to a few times per day
Kanera et al. (2016) [39] Randomized controlled trial Follow-up at: 3 months 6 months (end of intervention) 12 months (post-baseline)	Number of KNW modules followed on average by intervention participants: 2.23 (SD = 1.58). Following the PA module: *n* = 45 (24.73%) Following the diet module: *n* = 116 (61.70%) Following the smoking module: *n* = 19 (10.1%) Diet and/or PA module followed within 14 weeks by 80% of intervention participants		*Accessing the KNW* may increase moderate physical activity (PA) levels Statistically significant *increase in moderate PA* with 74.74 min Significant increase in *moderate PA levels among the PA module users* compared with non-PA module followers: *p* = 0.22, *d* = − 0.32 (not significant after multiple testing) *Among diet module followers*, a significant increase in consuming (not significant after multiple testing): -Fruit (*p* = 0.031, *d* = −0.12) -Fish (*p* = 0.045, *d* = −0.11) Significant intervention effect on *vegetable consumption at 6 months* (*p* = 0.27, *d* = − 0.37; intention-to-treat analysis (ITT): *p* = 0.023. All results not significant after multiple testing *Among diet module non-followers*, a significant increase in consuming (not significant after multiple testing): Vegetables: (*p* = 0.048, *d* = − 0.23) Smoking behaviours at 6 months: no significant intervention effects neither for complete cases (*p* = 0.233) or after ITT analysis (*p* = 0.278). 33% (*n* = 9) of the *n* = 27 smokers in the Intervention (IC) group quit smoking at 6 months 12.5% (*n* = 4) of the *n* = 32 smokers in the Usual Care (UC) group quit smoking at 6 months
Kanera et al. (2017) [36] Randomized controlled trial Follow-up at: 6 months (end of intervention) 12 months (post-baseline)	Usage: The PA module was used by *n* = 46 (28.1%) from the Intervention group at 12 months post-baseline	Moderation effects: Significant moderation effect found for age: KNW was more effective in participants younger than 57 years of age at 6 months (*p* = 0.040) and 12 months (*p* = 0.000) No significant moderation effect found for gender *(p* = 0.296) No significant moderation effect found for education level (*p* = 0.351) No significant moderation effect on PA outcomes found for using the PA module at 6 and 12 months post-baseline (at 6 months: *p* = 0.218; at 12 months: *p* = 0.480)	Effects of the KNW usage on moderate PA levels: 1) Between group statistical difference between IC group and UC group at 6 months: 78.3 min per week 2) Between group statistical difference between IC group and UC group after 12 months: 106.5 min per week 3) Statistically significant between-group differences in moderate PA levels at 12 months post-baseline (*p* = 0.010, *d* = 0.35, ITT: *p* = 0.011) Effects of the KNW usage on vegetable consumption at 6 and 12 months: Significant intervention effect on vegetable consumption at 6 months (*p* = 0.001, *d* = − 0.37, ITT: *p* = 0.002) No significant intervention effect on vegetable consumption at 12 months (*p* = 0.121, *d* = − 0.28, ITT: *p* = 0.132)
Lee et al. (2013) [46] A mixed-method qualitative and quantitative study	Findings from the evaluation phase: Usability: The end-users rated the intervention to be easy to use and understand—mean total usability score (SD) = 81.3 (20.2) points out of 100 Feasibility: Programme feasibility: 90% of the participants (*n* = 27/30) used the intervention consistently throughout the 12-week intervention period Cronbach alpha coefficient = 0.87	Findings from the development phase: Barriers to exercising: - Concerns whether being overweight increases cancer recurrence—not sure how to exercise during recovery or treatment periods - Not sure of what precautions are needed during exercising - Impaired routine due to cancer treatment - Lack of exercise partner - Inadequate exercise conditions (lack of local gyms, no parks nearby, bad weather - Side effects from cancer treatment (fatigue) - Lack of motivation to exercise - For females—difficult to exercise outdoors when dark Facilitators to exercising: - Encouragement from friends/healthcare professionals (HCPs) - Perceptions of increased energy levels and/or well-being increased motivation for exercising	Findings from the development phase: Healthy dieting habits: - Participants wanted more information on food to eat and to avoid. Barriers to fruit and vegetable consumption: - Lack of preparation time - Taste concerns - Fear of pesticide exposure - Conflicting information in media vs. research regarding dietary recommendations - Difficulty in self-motivation for healthy dieting behaviour - Busy lifestyle - Difficulty finding healthy foods in restaurants - High costs of fruit and vegetables - Fruit and vegetables quickly rotten
Lee et al. (2014) [40] A 12-week pilot randomized controlled trial with a control group Follow-up at: 12 weeks (end of intervention)	Programme feasibility and acceptability: 89% of the participants used the intervention consistently throughout the 12-week intervention period Significant *adherence* to the final exercise module Positive user evaluation of the WSEDI contents, the delivery mode via the Internet and intervention usefulness		Intervention effect on exercising: Statistically improved between-group difference in moderate exercising for ≥ 150 min per week in the WSEDI intervention group (*p* < 0.0001) Intervention effect on fruit and vegetable consumption: -Statistically significant improvement in the Intervention group in eating 5 F&V servings per day (*p* = 0.001) -Statistically improved overall dietary quality according to the DQI (*p* = 0.001) -Statistically higher proportion of participants in the Intervention group with protein intake according to RDA (*p* = 0.016) and in calcium intake (*p* = 0.003) HRQoL (statistically improved compared with controls): -Physical functioning (*p* = 0.023) -Appetite loss (*p* = 0.034) *Fatigue severity* according to BFI: Statistically improved compared with controls (*p* = 0.032) Stage of change according to the TTM: statistically improved in the Intervention group compared with the controls for *motivational readiness for*: -Exercising: (*p* < 0.0001) -F&V consumption (*p* < 0.029) Statistically significant between-group difference in *self-efficacy* for -Exercise management (*p* = 0.024) -Increased F&V consumption (*p* = 0.023)
Melissant et al. (2018) [41] A pre-test-post-test feasibility study Follow-up at: 1 week	Actual intervention usage: - Intervention used by 75% (57/76) of participants (including dropouts) - Intervention used by 84% (57/68) of participants (excluding dropouts) Satisfaction: - A mean score for satisfaction with Oncokompas = 6.9/10 - 77% (*n* = 44) of the users viewed the Learn module - 63% (*n* = 36) of the users read their self-care advice - 61% (*n* = 35) of the users read the Act module - 58% (*n* = 33/57) of the survivors used the BC module. Satisfaction with the BC module was 7.6/10 Net promoter score: negative (− 36) - Detractors: 46% - Promoters: 10% - Passives: 44%	Adoption (intentions to use the intervention: to survey filled in by 75% (76 out of 101 eligible participants) Satisfaction-associated factors: -Treatment with surgery + chemo/radiation therapy vs. surgery only (75% vs 25%) *p* = 0.013 Barriers and facilitators to usage: - Most common *barrier*: intervention too extensive - Most common *facilitator*: congruency of the well-being score generated by Oncokompas and the participants’ own perceived well-being (41%, 24/59 participants) Arm and shoulder movement section = *n* of times accessed by participants out of *n* = 57 (%): - Personalized information: 7/57 (12%) - Self-care advice: 5/57 (9%)	Patient activation measure (PAM): significantly higher after intervention use: *p* = 0.007, *r* = 0.24 (small effect size) Perceived efficacy in patient-physician interactions (PEPPI-5): not significantly improved after intervention use: *p* = 0.75
Myall et al. (2015) [45] An in-depth qualitative process evaluation study Follow-up at: 6 and 12 weeks post-baseline	Purpose of participating in the trial: - Majority of the participants found it beneficial to take part in the RCT resulting in behavioural and lifestyle changes in some participants after using the RESTORE intervention - Participants benefited from feeling supported during the trial - Learning, self-reflecting, realizing and acceptance of fatigue as a limitation Workload required: Most of the participants: - Did not need any new skills for using RESTORE - Found it easy to fit RESTORE into daily routine	Content of the RESTORE intervention: - Majority found the language of RESTORE accessible and not complicated - Some participants would prefer more tailored information with additional signposting resources Barriers to using the intervention in everyday life: - Intervention not relevant - Additional skills required to use the intervention - Difficulty fitting the intervention into daily routines - Unintended negative impact on participants reminding them of cancer and treatments Needed improvements of the implementation of the intervention in relation to: - Timing of accessing the intervention post-treatment - Modes of delivery of the intervention within the intervention (graphics, pictures) - Equality of access to the intervention Ways to improve the intervention implementation: To enhance the content of RESTORE, one participant suggested for more graphics and pictures to be added: - Providing equal intervention access based on the users’ socioeconomic status - Providing equal access to the intervention in terms of cognitive and mental status of the user	
Paxton et al. (2017) [42] Randomized parallel-group feasibility study At 3 months	Satisfaction with the intervention components: - No significant between-group differences - Both groups found most satisfying: tips for overcoming barriers and goal achievement, the goal setting tools and the health notes Overall satisfaction: - No significant between-group differences (*p* = 0.24) - PA group: 3.9/5 (1 = not satisfied at all, 5 = very satisfied) - Dietary group: 4.3/5 Website demand - No significant between-group differences - PA group average visit to the website: 9.6 out of 12 weeks - Dietary group average visit to the website: 10.7 out of 12 weeks, *p* = 0.15	*Likelihood of recommending* the intervention 97% of the completers would recommend the intervention to others *Likes/dislikes* about the ALIVE intervention The most “liked” components of the intervention: - Educational information (36%) - Email reminders (14%) - Goal setting tools (12%) - Ease of use (9%), motivation or encouragement (9%) The most “disliked” components of the intervention: - Functionality (48%) - Information (31%) - Tools (14%) - Time (7%) *Perceived effectiveness* of the ALIVE intervention in changing health behaviours - No significant between-group differences (*p* = 0.67) - PA group: 3.7/5 (1 = not satisfied at all, 5 = very satisfied) - Dietary group: 3.8/5	Minutes of moderate to vigorous exercising per week Statistical improvements in PA exercising/week for participants in both intervention tracks *p* < 0.001, *d* = 0.20 (ITT) 97 additional minutes of moderate PA/week for the PA and *p* < 0.001, *d* = 0.20 (ITT) 49 additional minutes of moderate PA/week for the dietary group *p* < 0.001 *d* = 0.20 (ITT) Sedentary behaviour (ITT): - Significant reductions in discretionary minutes (*d* = 0.20), other minutes of sedentary time per week (*d* = 0.15), television-related time per week (*d* = 0.15) and total sedentary time (*d* = 0.45 in both groups, *p* < 0.001) - Significant improvements in the PA group > than significant improvements in the dietary track (*p* < 0.001) - Total sedentary time in the PA group reduced with 304 mins/week—5 times greater reduction than in the dietary group (−59 mins/week, *d* = 0.45, *p* < 0.001) Dietary intake Within-group statistical improvement for the dietary group for F&V intake by + 0.7 cup servings per day (*p* = 0.002, *d* = 0.34. No statistical between-group differences neither in completers only, nor in the ITT analysis No other significant within-group improvements in dietary intake for neither the PA nor the dietary group
Willems et al. (2017) [37] Randomized controlled trial Follow-up at: 6 months (end of intervention)	Module use: - At 6 months on average 2.2 modules were used −89.4% of participants used at least 1 module - The Fatigue module was used by 37.2% of the participants - The Mood module was used by 24.5% of the participants		Intervention effects 6 months post-baseline: - Increased emotional functioning: (*p* = 0.022, *d* = 0.15) - Increased social functioning (*p* = 0.011, *d* = 0.15) - Decreased depression (*p* = 0.007, *d* = 0.21) - Decreased fatigue (*p* = 0.020, *d* = 0.21) With ITT analysis effects on *depression* and *fatigue* remained significant: - Effect on depression: *p* = 0.039 - Effect on fatigue: *p* = 0.019. *Influence of module use* (before multiple testing as no significant results after multiple testing): indications that module use influenced the intervention effects on: - Fatigue (if using 2 to 8 modules): *p* = 0.022, *d* = 0.28 - Depression (if *not* using the Mood module): *p* = 0.017, *d* = 0.27 - Social functioning (if using the Fatigue module): *p* = 0.009, *d* = 0.37

**Table 6:** Breakdown of quality appraisal scorings and inter-rater agreement kappa and weighted kappa values for the qualitative studies [30]

Study	Item on Kmet et al. checklist										Summary score	Kappa value	Weighted kappa value
	**1)** Question or objective sufficiently described?	**2)** Evident and appropriate design	**3)** Clear context for the study	**4)** Linked to a theoretical framework	**5)** Appropriate and detailed sampling strategy	**6)** Clear and detailed data collection methods	**7)** Complete, appropriate and systematic data analysis	**8)** Verification procedure(s) used in the study	**9)** Conclusions supported by results?	**10)** Evident reflexivity			
Harder et al. (2017) [44]	2	2	2	1	2	2	2	0	2	0	**0.75 (75%)**	**0.75**	**0.86**
Myall et al. (2015) [45]	2	2	2	2	1	2	2	0	2	1	0.80 (80%)	**0.36**	**0.56**

Scoring (Kmet et al., 2004) [30]: total sum = (number of “yes”*2) + (number of “partials”*1) Total possible sum = 20 Summary score: total sum/total possible sum

**Table 7:** Breakdown of quality appraisal scorings and inter-rater agreement kappa and weighted kappa values for the quantitative and mixed-methods studies [30]

Study	Item on Kmet et al. checklist														Agreed scores	Kappa value	Weighted kappa value
	**1)** Question or objective sufficiently described?	**2)** Evident and appropriate design	**3)** Subject selection	**4)** Subject characteristics	**5)** Random allocation	**6)** Blinding of investigators	**7)** Blinding of subjects	**8)** Defined and robust OMs	**9)** Sample size	**10)** Analysis described and appropriate	**11)** Estimate of variance	**12)** Controlled for confounding	**13)** Sufficient Results	**14)** Results match Conclusions?			
Cnossen et al. (2016) [43]	2	2	1	2	N/A	N/A	N/A	2	2	2	2	N/A	2	2	**0.95 (95%)**	**0.66**	**0.84**
Foster et al. (2016) [38]	2	2	1	2	2	2	N/A	2	2	2	2	2	2	2	**0.96 (96%)**	**0.46**	**0.73**
Kanera et al. (2016) [39]	2	2	2	2	2	0	N/A	2	2	2	2	2	2	2	**0.92 (92%)**	**0.72**	**0.87**
Kanera et al. (2017) [36]	2	2	2	2	2	0	N/A	2	2	2	2	2	2	2	**0.92 (92%)**	**0.72**	**0.87**
Lee et al. (2013) [46]	2	2	1	1	N/A	N/A	N/A	1	2	1	1	N/A	2	2	**0.75 (75%)**	**0.78**	**0.89**
Lee et al. 2014 [40]	2	2	2	2	2	0	0	1	2	1	2	2	1	2	**0.75 (75%)**	**0.30**	**0.49**
Melissant et al. (2018) [41]	2	2	1	2	N/A	N/A	N/A	2	2	2	2	N/A	2	2	**0.95 (95%)**	**1.00**	**1.00**
Paxton et al. (2017) [42]	2	2	2	2	2	N/A	N/A	2	2	2	2	2	2	2	**1 (100%)**	**0.77**	**0.90**
Willems et al. (2017) [37]	2	2	2	2	2	0	N/A	2	2	2	2	2	2	2	**0.92 (92%)**	**0.72**	**0.87**

Scoring (Kmet et al., 2004) [30]: total sum = (number of “yes”*2) + (number of “partials”*1) Total possible sum = 28 − (number of “N/A”*2) Summary score: total sum/total possible sum

### Analysis and synthesis of the results

A narrative approach [35] was used to synthesize study characteristics and key findings of the included evidence. The included studies were categorized and agreed on as quantitative, qualitative or mixed-methods studies by two of the reviewers, based on the study design definitions presented by the study authors and on the type of their quantitative, qualitative or mixed findings [35].

## Results

A total of 696 records were identified and 41 underwent full-text review. Eleven studies published between 2013 and 2018 and with a total sample size (*n* = 965) met the study inclusion criteria and were included in the synthesis. Five studies were conducted in the Netherlands, three in the United Kingdom, two in South Korea and one in the United States. There were three randomized controlled trials (RCTs) which were presented in five different studies [36–40] (with predominantly quantitative study designs [36, 37, 39, 40] and one mixed-methods RCT and process evaluation study [38]), three feasibility studies, all with quantitative study designs [41–43], one qualitative early user testing study [44] and two evaluation studies [45, 46] that had qualitative [45] and mixed-methods [46] study designs. The studies with quantitative designs that constituted the majority of all studies in this review (*n* = 7) used single-group feasibility design [43], an RCT [36, 37, 39], a pilot RCT [40], a pre- and post-test feasibility study [41] and a randomized parallel-group feasibility study [42]. The two studies with the entirely qualitative designs and qualitative findings [44, 45] were conducted using focus groups [44] and in-depth interviews [45] for the purposes of conducting qualitative testing [44] and an evaluation [45] of their interventions. The two studies that adopted mixed quantitative and qualitative methodologies [38, 46] conducted process [38] and formative [46] evaluations and, hence, provided both types of data, with prevailing quantitative data in them.

The most commonly used tools for quantitative data collection across the studies were study-specific surveys or questionnaires, validated outcome-specific tools which occasionally were adapted and/or translated into the participants’ language, semi-structured telephone interviews, data usage, standard questionnaires and self-reported questionnaires. The collection of qualitative data was mainly performed using telephone interviews, open-ended questions and an evaluation survey. Lee et al. [46] in their study used qualitative semi-structured interviews to obtain their qualitative data during the intervention development and questionnaires with 7-point scales in order to obtain quantitative data for process evaluation.

### Demographic characteristics of included studies

Sample sizes (total *n* = 965) varied greatly: from 13 participants in one qualitative study [44] to 462 participants in a randomized controlled trial (RCT) described in three articles [36, 37, 39]. The sample size range within the qualitative studies [44, 45] was much smaller (*N* = 13 and *N* = 19, respectively) than the sample sizes in the studies with quantitative designs. However, even within the studies with quantitative designs, variations depending on the type of study were noted. The three quantitative feasibility studies [41–43] had relatively smaller sample sizes of *N* = 68, *N* = 71 and *N* = 38, compared with the significantly larger sample sizes within the RCT studies with samples of *N* = 462 [36, 37, 39] and *N* = 159 [38]. Noticeably, the pilot RCT study by Lee and colleagues [40] also had a relatively small sample size (*N* = 59) compared with the other RCTs included in this review.

Participants across seven out of the 11 articles (described in detail in Table 3) were predominantly females (Median: 80%, IQR: 20%), with two studies [40, 44] having entirely female populations. The only study with a male majority of participants was by Cnossen et al. [43], where 76% were men. Three articles [41, 42, 46] did not explicitly report the gender of their participants. Participants across all featured studies had a mean age of 53.2 years with the youngest participants with a mean age of 41.5 years [46] and with the oldest participants’ mean age of 65 years [43].

The most prevalent type of cancer diagnosis that the participants had was BC. In five out of 11 studies [40–42, 44, 46], all participants had BC and received various types of breast surgeries (see Table 3). Only one study [43] had participants who all had a cancer different to BC (laryngeal cancer) and they received head and neck (HAN) surgery. The studies by Foster et al. [38] and by Myall et al. [45] included participants with, respectively, seven and five differing types of cancers (with the relevant surgeries). BC was again the most prevalent one. Kanera et al. [36], Kanera et al. [39] and Willems et al. [37] reported on the same RCT participant sample, the majority (70.5%) of whom had BC. Four studies [36, 37, 39, 41] had imposed a minimum of 4 weeks since surgery or other treatment as inclusion criterion. Three studies [38, 40, 45] had no minimal time threshold since surgery or treatment. Paxton et al. [42] and Lee et al. [46] had no upper time limit since initial cancer diagnosis or treatment, whereas Foster et al. [38] and Myall et al. [45] set a 5-year maximum period since diagnosis for inclusion. All participants, except for those in the studies by Harder et al. [44] and Lee et al. [46], were not receiving or had completed radiotherapy and/or chemotherapy treatments. Cnossen et al. [43] did not exclude the presence of radiotherapy and/or chemotherapy treatments but did not report participants undergoing such treatment.

### Intervention characteristics

The 11 articles analysed in this review described seven individual interventions: (1) Cnossen and colleagues [43] described the “In Tune without Cords” (ITwC) intervention; (2) Foster et al. [38] and Myall et al. [45] described the RESTORE intervention; (3) Harder and colleagues [44] described the bWell app intervention; (4) the Kanker Nazorg Wijzer (KNW) intervention or, in English, the “Cancer Aftercare Guide” intervention was reported by Kanera and colleagues [36, 39] and by Willems and colleagues [37]; (5) Lee and colleagues [40, 46] described the web-based self-management exercise and diet intervention (WSEDI); (6) Melissant et al. [41] described the Oncokompas intervention; (7) Paxton et al. [42] described the ALIVE intervention. These seven interventions described across 11 articles included five web-based interventions described in nine articles [36–41, 43, 45, 46], a mobile app [44] and an intervention sent by email and web-based [42]. Four out of the 11 selected articles reported on their own individual interventions [41–44]. Four studies had no comparator group and were single-group studies [41, 43, 44, 46]. Three studies adopted usual care as their comparator intervention [36, 37, 39], whereas three studies compared their online intervention to using a leaflet [38, 40, 45] and one study [42] had two interventional groups and compared the uptake of the two different “tracks” of their intervention.

Each of the seven interventions in this review addressed a range of different domains as follows: (1) Cnossen et al. [43] (nutrition; tracheostomy care; voice prosthesis care; speech rehabilitation; smell rehabilitation; and mobility of the head, neck and shoulder muscles); (2) Foster et al. [38] and Myall et al. [45] (cancer-related fatigue, goal setting, diet, sleep, exercise, addressing issues around home and work life; thoughts and feelings; and talking to others); (3) Harder et al. [44] (self-management of arm and shoulder exercises); (4) Kanera et al. [36, 39] and Willems et al. [37] (physical activity, diet, smoking cessation, return to work, fatigue, anxiety and depression, social relationship and intimacy issues and general information on the most common residual symptoms of cancer); (5) Lee et al. [40, 46] (self-management of exercise and diet); (6) Melissant et al. [41] (endocrine therapy, (early) menopausal symptoms, body image, fertility issues, hereditary breast cancer, lymphoedema, fibrosis, arm-shoulder movement, breast reconstruction, breast prosthesis and sexuality); and (7) Paxton et al. [42] (behaviour change regarding physical activity or diet).

All interventions but one [44] were Internet web-based and participants accessed these via a web browser. Only the intervention by Harder et al. [44] was a downloadable mobile application. The intervention periods varied: the shortest being 1 week for intervention usage [41] and the longest being 6 months described by Kenara et al. [36, 39] and by Willems et al. [37]. The most common intervention duration was 12 weeks long, which was noted across the two interventions by Lee et al. [40, 46] and by Paxton et al. [42].

Topic-wise, one intervention aimed to raise participants’ general awareness and knowledge about cancer, its treatment and supportive services [41] and another intervention [43] provided specific advice about laryngeal cancer and its aftermath. Harder et al. [44] designed their intervention specifically for upper limb exercising after BC surgery. The single intervention “RESTORE” that was written up in the two studies by Myall et al. [45] and by Foster et al. [38] was specifically about coping with fatigue. The most common combination of topic modules was about a healthier diet and increased levels of physical activity (PA) included across two of the interventions by Kenara et al. [36, 37, 39] and by Lee et al. [40, 46]. Table 4 presents a breakdown of all the features of the interventions across the studies and their duration.

Table 4 presents a breakdown of the intervention features. Many of the articles reported common intervention features, for instance, all interventions included password-restricted login access, specific or non-specific exercise programmes or advice and images and visual graphics. All but one [43] offered automated and individually tailored progress feedback notifying the intervention user of achieved goals and self-regulation purposes while using the interventions; for instance, they provided personalized feedback on dietary behaviours, as per pre-set goals in the intervention by Kanera et al. [36, 39]/Willems et al. [37]. The two interventions described by Foster et al. [38]/Myall et al. [45] and by Cnossen et al. [43] did not offer tailored educational information and online self-evaluation of progress, unlike the other interventions in this review. The offered tailored educational information was usually provided by automated personalization of the information for advice and educational purposes, depending on the user information provided prior to or during using the intervention and aiming to correspond to their needs, for instance, the tumour-specific BC educational information for intervention users who have had BC [41]. The features for self-evaluation of progress while using the intervention were usually tools allowing self-ticking options for self-monitoring purposes within the intervention [42] or for self-reporting to the research team web-based progress outcomes in the form of surveys [40, 46]. Other features were printable results [41], automated phone calls with a coaching session and achievement rewards [42], automated telephone text messages [40, 46], a “frequently asked questions” section [44], video animations [43] and videos with healthcare professionals and/or educational information and advice [36, 37, 39]. The interventions by Foster et al. [38], Paxton et al. [42] and Myall et al. [45] released their contents weekly. Foster et al. [38], Harder et al. [44], Lee et al. [40, 46] and Myall et al. [45] involved the use of a diary. Additional information for signposting was provided in most interventions: Foster et al. [38], Harder et al. [44], Kanara et al. [36, 39], Melissant et al. [41], Myall et al. [45] and Willems et al. [37].

### Quality assessment and inter-rater reliability

All included studies were rated as having “good” or “strong” methodological quality. The overall qualitative and quantitative combined quality scores ranged from 75 to 100% (median score: 92%, IQR: 17.5%). Tables 6 and 7 show the quality scorings for each criterion for all qualitative and quantitative design studies, respectively, and the inter-rater levels of agreement. A substantial level of agreement between the assessors on seven of the 11 included articles was achieved. Adjusting the calculations with weighted kappa values, the raters achieved “almost perfect” agreements on seven of the 11 articles, a substantial agreement on one article and a slightly lower, but moderate agreement on two articles (Tables 6 and 7).

Two articles [44, 45] were assessed with the qualitative checklist and achieved scores of 75% (implying good methodological quality,) and 80% (implying strong methodological quality), respectively. Both articles fully satisfied six out of ten quality criteria (Table 6). However, neither of the articles presented evidence of verification procedures in order to support the credibility of their qualitative results.

Nine articles [36–43, 46] were assessed with the quantitative checklist and achieved quality scores that ranged between 75% and 100% (median score: 92%, IQR: 12%). Table 7 shows that all articles have achieved scorings indicating “strong” methodological quality (> 80%), apart from the two articles by Lee and colleagues [40, 46] which were categorized as having a “good” methodological quality. All articles achieved maximum scores on four of the criteria. In all but one RCT [40], the nature of the study designs precluded subject blinding and that criterion was marked as non-applicable. Although participant blinding was deemed as being possible and attempted in Lee et al. [40] by not informing the participants whether they were allocated to the interventional or to the control groups, there was no evidence that this was achieved. This is so since the authors acknowledged that some of the participants might have guessed that the WSEDI intervention was the one being tested [40]. The study design allowed possible blinding of the investigators in five of the articles [36–40]; however, only Foster et al. [38] presented evidence for appropriate investigator blinding procedures. One article [46] failed to report the recruitment process and the gender of their participants.

### Main outcomes of interest

The three main outcomes of interest (adherence and usage, acceptability and satisfaction) were analysed across all included 11 studies, as long as these were present in them, irrespective of the type of methodology and findings that these studies possessed: quantitative, qualitative or mixed quantitative and qualitative. The findings concerning adherence and usage were analysed in all studies, except for the qualitative only study by Myall et al. [45] as this outcome was not described in it. The outcomes for acceptability were described in one of the two studies with mixed qualitative and quantitative methodologies and findings [38], also in both qualitative studies [44, 45] and in one out of the seven quantitative studies [40].

### Adherence and usage

Adherence to the interventions was measured and described in all articles, except for Myall et al. [45]. Predominantly, this was achieved by tracking login and usage data or self-reported measures (Table 5). Adherence levels across the included articles were generally high, but the longer the intervention period and follow-up lasted, the lower the adherence levels were. Follow-up periods varied between 1 week [41] and 12 months in Kanera et al. [36]. Adherence was mainly measured in percentages and varied between 10.1% at 6 months [39] to 100% for at 8 weeks in Harder et al. [44]. Most studies had predefined cut-off levels of adherence [36–42]. Foster et al. [38] considered participants as adherent if at least two out of five modules were accessed; Kanera et al. [36] required at least three pages accessed within each module for adherence.

### Acceptability

Acceptability was measured in four studies [38, 40, 44, 45] describing three interventions. Based on the provided outcomes for acceptability across the four studies that measured it, the majority of the participants across these studies had positive feedback and opinions of the interventions they were using, finding the interventions acceptable and beneficial, which led to positive behaviour and lifestyle changes (Table 5). Foster et al. [38] measured acceptability by exploring participants’ perceptions of the intervention timing, the attrition rate (36%), identified benefits from participation, adherence levels (71%) and preferred mode of access (50% preferred using the RESTORE intervention along with a leaflet). Harder et al. [44] measured acceptability of their intervention by exploring its usability and attractiveness during a focus group discussion, whereas Lee et al. [40] measured the participation in the programme during the interventional period (89%). The level of acceptability was determined through telephone interviews in Myall et al. [45], where the authors found that participants benefited from using the intervention, and this resulted in a positive lifestyle behaviour change for the majority of their participants.

### Satisfaction

Satisfaction with the intervention was reported in three articles [41–43] using different outcome measures (Table 5) and was predominantly positively evaluated by intervention users. Only in Melissant et al.’s [41] satisfaction was negatively reported: net promoter score (NPS) was negative at − 36 (range: − 100 to + 100 describing how many of the intervention users would promote it to others (if more than the anti-promoters, then this is considered positive”, how many would not promote it to others and how many would take a passive stance and would neither promote it). Apart from this, their other satisfaction outcomes were positive: mean score for satisfaction with the intervention was 6.9 out of 10 and with the specific BC module—7.6 out of 10. The “Learn”, the “Self-care advice” and the “Act” modules were all viewed by more than 50% of the participants. Cnossen et al. [43] measured satisfaction with the overall intervention (84%), user-friendliness (74%), overall satisfaction (66%) and a net promoter score (NPS = +5). Paxton et al. [42] used a 5-point Likert scale to measure overall satisfaction and satisfaction levels with the intervention components. They also found that 97% of their respondents would recommend the intervention with the most popular component being the “Educational Information” and the least popular component being “Functionality”. However, no significant between-group differences regarding overall satisfaction were found.

### Secondary outcomes of interest

#### Moderating factors and associations affecting adherence, acceptability or satisfaction

Moderating factors and associations affecting intervention adherence, acceptability or satisfaction were reported, respectively, in two studies [36, 43]. Kanera et al. [36] found that younger participants (age < 57 years) used the intervention significantly more which proved that younger age, unlike gender, education level and use of the physical activity (PA) module, positively affected intervention use at 6 months (*p* = 0.040) and at 12 months (*p* = 0.000). This effect of moderation was also confirmed by secondary analyses. Conversely, Cnossen et al. [43] did not conduct analysis that assesses moderations; however, they found a statistically significant positive association between satisfaction with their intervention and education level (*p* = 0.004) and also for health literacy skills positively affecting satisfaction levels (*p* = 0.038)—i.e. the higher the levels of educational level and health literacy skills, the higher levels of satisfaction with the intervention.

### Other outcomes of interest

#### Barriers and facilitators to intervention usage

Barriers and facilitators to intervention usage and adherence were explored by Lee et al. [46], Melissant et al. [41] and Myall et al. [45] through (telephone) semi-structured interviews and surveys. *Barriers* were identified as the intervention being too extensive [41] and having lack of time, new skills needed and negative impacts from cancer memories [45]. Common *facilitators* for usage were when the scoring for well-being generated by the intervention was similar (41%) to participants’ own perceptions [41], and accessible, easy-to-understand language was used within the intervention [45]. Lee et al. [46] also measured *perceived ease of use* and reported that their intervention was perceived as easy to use and understand, with a mean usability score of 81.3/100 (SD = 20). Paxton et al. [42] found no significant between-group difference for another self-reported outcome, *perceived effectiveness of the intervention*, which meant that the PA and the Diet groups who used the online intervention perceived it to be similarly effective (Table 5).

#### Suggestions for improvement

Suggestions for improvement of interventions were requesting additional demonstration videos or sections including frequently asked questions [44], more specific information, precautionary advice [46], quicker access to the intervention postoperatively, improved intervention interface and equal opportunities to access the intervention regardless of social, economic and geographical factors [45].

## Discussion

The aim of this review was to evaluate the current literature and explore adherence, acceptance and satisfaction with Internet self-management interventions for cancer rehabilitation after surgery and whether intervention features or other factors affected these outcomes. The studies reported in this review were classified as having “good” to “strong” methodological quality. Evidence was provided that participants were more inclined to be satisfied with, to accept and adhere to the interventions if the following criteria were present: the intervention was time and cost-efficient, required the acquisition of minimal or no new skills, was presented with coherent language, was offered as soon as possible after cancer treatments and contained the essential precautionary and educational information relevant to and tailored for the individual user. These findings are supported by another systematic review of web-based interventions for symptom management in cancer patients by Fridriksdottir et al. [47]. These authors reported that web-based interventions can have a positive effect on cancer symptoms management provided that the interventions are timely and include evidence-based information, tailored feedback and self-management components.

There was a wide range of adherence to interventions that varied across the studies. Analysis showed that adherence was significantly better where contents had been personally chosen by the users [42], interventions with personalized information [40, 43, 45] and interventions with tailored information [41, 44]. A similar wide range of adherence to healthcare web-based interventions, directly correlated to the intervention duration, was also noted by Kelders et al. [26] in their systematic review. Results showed that all interventions with adherence above 80% lasted between 6 and 21 weeks, and with average adherence levels to these interventions of 55%, whereas interventions with durations ranging between 52 to 130 weeks had an average adherence level of 39%.

Kelders et al. [26] also correlate web-based intervention adherence to its “intended usage”, i.e. to the recommended by the intervention creators “extent” of usage for gaining maximum benefits from the treatment intervention. However, only a few studies mention intention to use the intervention in some form. Lee et al. [40] mention “intended usage” in their study and the fact that they have provided the information about the intended usage of the intervention to their study participants in the form of a manual containing recommended optimal for the user dietary or exercise parameters. Kanera et al. [36] mention the “intended action” as a feature in their action planning component, referring to a specified action to be done by the participants, in order to perform a given behaviour change. Melissant et al. [41] have predefined the feasibility of their intervention as 50% or more adoption and usage “as intended, based on login data”, however, not providing a clear description of what the intended usage as per login data was. Another study in this review provided recommended cut-off rates of usage based on physical activity and dietary guidelines in the field of the intervention [42]. As per the definition provided by Kilders et al. [26], one could argue that the intended usage of an intervention can match the predefined cut-off usage levels by most of the authors that measured adherence in this review and predefined these. However, some of the studies did not provide an accurate description of the predefined intended cut-off usage levels or a rationale for the predefined usage levels [37], and whether or not these predefined usage levels were expected to bring more benefits to the user compared with less usage of the intervention [36, 39]. Some studies did not specify any predefined cut-off usage levels at all [44, 46]. The last two comments imply a potential knowledge gap showing the need for intervention designers to explicitly describe the intended usage of an intervention rationalizing the benefits that the intended levels of usage would bring to the intervention user.

Education level was reported to be a moderating factor for intervention adherence with significant correlation between the two described by Cnossen et al. [43] and with non-significant correlation between education level and adherence reported by Kanera et al. [36]. When considering the results of the two studies, it should be noted that the sample size in the RCT by Kanera et al. [36] was nearly ten times larger than the sample by Cnossen et al. [43]. Moreover, only Kanera et al. [36] analysed their data based on the intention-to-treat (ITT) principle which generally provides a less biased estimation of treatment effectiveness [48] and thus makes the results of this study more reliable than others [48]. Also related to the reliable reporting of study samples, failing to report the recruitment process and the gender of their participants, as did the authors in Lee et al. [46], is a major flaw in this article. Poor recruitment reporting can also inflict significant risk of bias and thus reduce the quality of a study [49].

Three interventions described by Cnossen et al. [43]; Kanera et al. [36, 39]/Willems et al. [37] and by Paxton et al. [42] were based on a theoretical rationale, and no public or patient involvement in the designing of these interventions was described which suggests that these authors may have omitted the inclusion of features or components that the intervention users would have liked or preferred. For instance, based on their users’ qualitative feedback, Harder et al. [44] added a “Frequently Asked Questions” option, a symptoms diary and tailored information sections, and they paraphrased some of the wording so that the intervention better reflected the users’ preferences. As the authors noted, this could have improved the user testing feedback and adherence to their intervention.

In terms of satisfaction, two studies had an opposing NPS: a positive NPS by Cnossen et al. [43] by head-and-neck (HAN) cancer survivors, versus a negative NPS by Melissant et al. [41] by their BC survivors. Since satisfaction levels have been correlated with adherence levels, exploring satisfaction can provide useful insight to researchers in terms of improving patients’ adherence to Internet-based interventions [28].

No studies reported any negative feedback related to acceptability of Internet interventions. A study by Short et al. [50] specifically looked into different delivery schedules of their intervention. Although this study was not included in this review due to unclear surgical status of their participants, it interestingly showed that an Internet intervention delivered monthly over 3 months was more acceptable for the participants compared with weekly delivered modules or as a one-off interventional episode. Moreover, Ryhanen et al. [51], in their systematic review of educational Internet or interactive computer-based interventions, aiming to increase the patients’ information and awareness of breast cancer and its symptoms, also synthesized some results showing that spending more time using the interactive interventions increased the information competence of the intervention users, compared with spending less interaction time. However, the authors of this review pointed out that the majority of the interventions included were delivered as a one-off session, instead of an intervention on a continuous basis. This therefore imposes questioning regarding the positive finding about spending more time with the interventions, implying that this finding was not a common observation, but rather a one-off finding from one of the studies included in that review. There is therefore a need for more research in the field of mode of delivery of the interventions in terms of duration and frequency of interaction with such web-based health-related interventions.

In terms of the outcomes describing *suggestions for improvements*, four out of seven interventions—the RESTORE intervention [38, 45], the bWell intervention [44], the Oncokompas intervention [41] and the WSEDI intervention by Lee and colleagues [40, 46]—involved people with cancer during their design and development stages, as recommended by the clinical guidelines for healthcare decision-making by Nilsen et al. [52] and other authors in the field of Internet health intervention design [18, 53, 54]. However, within this review, no correlation between the cancer patients’ involvement in the intervention designing and improved outcomes for intervention use was found.

### Limitations

This review has some limitations. Firstly, it included only studies published in English, which may potentially discount other valid studies that may have been published in another language. Secondly, although this review aimed to focus only on interventions for postoperative exercising and rehabilitation, some of the participants included across the studies (one-third or less from each study sample) had not undergone surgical treatment. However, the studies were deemed appropriate for inclusion as the majority of their participants had undergone surgical treatment for cancer.

### Strengths and implications for research

This review was based on thorough and systematic searches and included independent reviewers screening the selected articles and assessing the quality of the final selection. Identification of common positive intervention features and components will facilitate developers to build future Internet interventions that will improve the provision of rehabilitation services for cancer survivors, the majority of whom receive surgery after diagnosis and deal with its consequences afterwards.

## Conclusions and recommendations

Based on studies with good to strong methodological quality, this review provides evidence suggesting that Internet self-management interventions for postoperative cancer rehabilitation can be satisfactory, acceptable and usable, as long as:

- They contain tailored, succinct information.
- They are written in coherent and plain language.
- No or minimal new skills are required.
- They do not take excessive time to complete.

Due to the scarcity of RCTs, the findings from this review should be treated with caution. Despite no limitations on publication year being set, the short publication span of 5 years indicates the lack of accumulated empirical evidence regarding these novel interventions. This implies the need for future more rigorous, large-scaled clinical trials to be conducted in this area.
